# Prevalence, Morphology, and Risk Factors of Mandibular Ramus Fractures: A Retrospective Cohort Study

**DOI:** 10.1002/cre2.70458

**Published:** 2026-10-29

**Authors:** Stefan Krennmair, Simon Kienberger, Matthias Angerer, Lukas Postl, Gerald Krennmair, Michael Malek

**Affiliations:** ^1^ Department of Oral and Maxillofacial Surgery Kepler University Hospital Linz Austria; ^2^ Medical Faculty Johannes Kepler University Linz Linz Austria; ^3^ Department of Oral and Maxillofacial Surgery Ludwig Maximilian‐University Munich Munich Germany; ^4^ Department of Prosthodontics Sigmund Freud Private Universitiy Vienna (SFU) Vienna Austria

**Keywords:** mandibular ramus fracture, morphology, prevalence, risk factors

## Abstract

**Objectives:**

Because mandibular ramus fractures represent a rare entity among the various fracture patterns of the mandible, the present study evaluated the prevalence, morphology, and risk factors associated with mandibular ramus fractures.

**Material and Methods:**

The prevalence of mandibular ramus fractures was evaluated in patients diagnosed with mandibular fractures over 5 years. Along with epidemiologic data, fracture morphology, treatment modalities, and patient‐related risk factors, such as gonial angle and third molar impaction, were assessed for the ramus fractures and compared with angle fractures.

**Results:**

Out of 547 patients diagnosed with mandibular fractures, 15 (2.7%; 10 m/5 f) presented with ramus fractures. Patients with mandibular ramus fractures demonstrated a significantly higher rate of female patients (33.3% vs. 9.6%, *p* = 0.021) and multiple fracture patterns (66% vs. 40.9%, *p* = 0.042; OR 2.91) compared to those with angle fractures (115/547; 21%; 104 m/11 f). Regarding fracture morphology, 12/15 (80%) ramus fractures exhibited a vertical/oblique fracture line, while comminuted fractures were observed only in 3/15 (20%) patients. Road traffic accidents (RTAs) were the predominant etiology for ramus fractures (46.6%, *p* < 0.025) and served as a potent predictor for complex multiple fractures (OR = 6.48, *p* < 0.001). Although deeply impacted third molars were significantly less frequent in ramus fractures than in angle fractures (13.3% vs. 38.3%; *p* = 0.049), the mean gonial angle was wider in the ramus group (130.3° ± 7.2° vs. 127.4° ± 6.2°, *p* = 0.098). In contrast to maxilla–mandibular fixation (13.3%), open reduction and internal fixation (ORIF) was the primary treatment modality (86.4%, *p* < 0.01); both yielded a high success rate without severe complications.

**Conclusion:**

A high gonial angle and the absence of deep third molar impaction structurally predispose the mandibular ramus to injury, particularly acting as predictors for complex fracture patterns during road traffic accidents (RTAs).

## 1 Introduction

The mandible represents the largest and strongest bone of the face and, subsequently, due to its prominent position, it is frequently exposed to trauma, accounting for twice the number of midface fractures (Ellis et al. 1985; Agarwal et al. 2017; King et al. 2004). A wide variability of fracture patterns exists, which might be related to epidemiologic factors such as geographical area, socioeconomic status, and cultural aspects (King et al. 2004; Sakr et al. 2006; Boole et al. 2001; Subhashraj et al. 2007) as well as to the influence of biomechanical mandibular properties. In this context, the fracture pattern of the mandible is evidently influenced by mandibular position during injury inclusive as well as by the overlying soft tissue, as well as the presence/absence of teeth present. In addition, variations in force application in the anterior or lateral direction and intensity consequently result in symphyseal, parasymphyseal, and condylar fractures or in angle, body, and contralateral condylar fractures (Subhashraj et al. 2007; Barde et al. 2014; Bither et al. 2008).

According to the findings of numerous studies, the most common sites of mandibular fractures are located in the condyle (29.1%), mandibular angle (24.5%), symphysis (22%), and body (16%) region (Olson et al. 1982; Bonitz et al. 2025; Agarwal and Mehrotra 2020). Conversely, among the various anatomical subsites, the incidence of coronoid, alveolar, and ramal fractures is reported to be rare, where mandibular ramus fractures rank as the third least common fracture (Olson et al. 1982; Bonitz et al. 2025). Although there exists a range of reported incidence of mandibular ramus fracture being influenced by high population densities, as seen in India internationally, the ramus fractures account for approximately 2%–3% of cases in the United States, Germany, and Australia (King et al. 2004; Boole et al. 2001; Barde et al. 2014; Olson et al. 1982; Bonitz et al. 2025).

Anatomically, the ramus is structurally located between the subcondylar and mandibular angle region in a non‐dentate area(Olson et al. 1982; Bonitz et al. 2025). According to a previous study by Agarwal and Mehrotra (2020), fractures of the mandibular ramus can be subdivided into five classes based on different fracture lines. Essentially, these fracture lines pass through the ramus areas, predominantly running either obliquely from the sigmoid notch or from the coronoid process to the posterior/lower border of the mandible, or running horizontally from the anterior border to the posterior border of the mandible. Furthermore, comminuted fractures extending both vertically and horizontally are characterized by a fracture localization in the center of the ramus (Agarwal and Mehrotra 2020).

It is well known that the mechanism and origin of injury correlate with specific anatomical fracture patterns and locations within the mandible (Agarwal and Mehrotra 2020). Consequently, a crash or fall with an anterior force application and tension transmission results in symphyseal, parasymphyseal, and condylar fractures (Agarwal and Mehrotra 2020; Daniel et al. 2021; Krennmair et al. 2020). Conversely, direct lateral impacts will typically result in angle, body, and contralateral condylar fractures (Jadhav et al. 2015; Kanta et al. 2018). Although clinical symptoms of ramus fractures include the presence of pain, swelling, and trismus, the overlapping pterygomasseteric sling minimizes the displacement of ramus fragments after fracturing. In this context, the non‐displacement of fracture fragments remains a strong argument for the use of conservative closed treatments modality as maxillomandibular fixations (Kanta et al. 2018; Lee 2017). However, open access and surgical stabilization, which are often preferred by clinicians, have also been reported with a high clinical outcome success rate and patient satisfaction (Kanta et al. 2018; Lee 2017; Duan and Zhang 2008; Alfertshofer et al. 2025).

Concerning factors affecting mandibular angle fractures, numerous studies have reported that individuals with a high gonial angle along with impacted mandibular third molars are at an increased risk (Duan and Zhang 2008; Soos et al. 2024). However, there still exists a lack of information evaluating how radiographic features such as third molars and the gonial angle might influence the occurrence and formation of mandibular ramus fractures.

The aim of the present study was to evaluate the prevalence of mandibular ramus fractures and to classify them according to a proposed system. In addition, the current retrospective analysis aimed to evaluate radiographic measurements and anamnestic causalities affecting the influence and generation of mandibular ramus fractures.

## 2 Materials and Methods

### 2.1 Patients—Study Design

A retrospective observational chart analysis was conducted using the medical records of maxillofacial trauma patients presenting mandibular fractures at the Department of Oral and Maxillofacial Surgery, Kepler Klinikum Linz. The present study was conducted according to the principles embodied in the Helsinki Declaration for biomedical research involving human subjects. The study protocol was approved by the local ethics committee and was self‐funded by the authors and their institutions. The study has been written according to the Preferred Reporting Items for Observational Studies using the STROBE guidelines.

During a 5‐year period, from May 2020 through May 2025, eligible patient files with a diagnosed mandibular fracture were screened, with a special focus on the presence of mandibular ramus fractures serving as the study group population (SG). For the present study, ramus fractures (SG) were defined as those with fracture lines situated between the subcondylar region and the mandibular angle in the non‐dentate region. In addition, a control group (CG) consisting of patients with mandibular angle fractures was assessed to enable comparison of epidemiologic and radiographic measurements between SG and CG.

### 2.2 Epidemiologic and Patient‐Related Factors

From the patients' charts, epidemiologic data (age/sex) as well as anamnestic information regarding etiology were collected for both mandibular ramus and mandibular angle fractures. In this context, for all patients, the cause of the injury was assigned as follows: (a) road traffic accidents (RTAs), (b) violence, (c) falls, as well as (d) accidents (at household/sports). By using preoperative radiographs (orthopantomograms; CT), mandibular ramus and mandibular angle fractures were subdivided into isolated fractures and multiple/complex (comminuted) fractures.

At the initial clinical examination, the existing symptoms of mandibular ramus fractures were evaluated and rated as (a) pain, (b) swelling over the fractured region, (c) trismus, as well as features of an (d) open bite situation.

### 2.3 Radiographic Measurements

By using radiographic measurements (Orthopantomography, Computer tomography, CBCT) and applying the criteria defined by Agarwal and Mehrotra (2020), a specific classification of the ramus fractures was assessed as follows: Type I: Vertical/oblique fracture line extending from the sigmoid notch to either the inferior border or angle of mandible. Type II: Vertical/oblique fracture line extending from the coronoid process to either the inferior border or angle of the mandible. Type III: Horizontal fracture line extending from the anterior border to the posterior border of the ramus of the mandible. Type IV: Oblique fracture line extending from posterior border of ramus to inferior border of mandible (separating the angle segment). Type V: Comminuted fracture of the ramus of the mandible (may cause isolated fractures of the coronoid, condyle, and the angle of mandible).

Additionally, by using orthopantomography, the presence or absence of third molars as well as the value of the gonial angle was evaluated. When the third molars were present, their location was initially categorized according to the Pell and Gregory classification (PGC) (1933). Furthermore, by using a simple subdivision, the third molar classification was also recorded as non‐present, erupted, or deeply versus non‐deeply impacted, with a subsequent subdivision based on whether the third molar status might affect or not affect the mandibular ramus and angle for fracturing. In accordance with the findings of the study by Soos et al. (2024), the PGC AI, AII, and BI were rated as erupted and not affecting the fractures and were classified as similar to non‐presence of third molar (non‐affecting/non presence). In contrast, Pell and Gregory classification to CI‐III, AIII, BII, and BIII represented deeper impaction and were rated as having a greater influence affecting on ramus and angle fractures. The difference in prevalence, as well as the influence of third molar impaction on fractures, was compared for mandibular angle and ramus fractures (Soos et al. 2024; Pell and Gregory 1933).

The radiographic measurements of the mandibular gonial angle were also assessed to evaluate its influence on mandibular ramus and angle fractures. In this context, the mandibular gonial angle was measured by drawing a tangent to the posterior border of the ramus (articular–gonion) and tangent to the lower border of the mandible (gonion–gnathion). The determined values of the gonial angle were compared between the study group (*n* = 15) and the control group (*n* = 115).

### 2.4 Treatment Modality—Clinical Outcome

Considering the therapeutic approach, treatment of mandibular ramus and angle fracture was performed either by a conservative method using maxillomandibular fixation (MMF) or by open reduction with internal fixation (ORIF) and intermediate MMF. For all patients included in the ORIF procedure, a combined transoral and trocar‐assisted transbuccal approach was used for miniplate positioning and osteosynthesis scew fixation. When ORIF was performed, MMF was initially used to stabilize the fracture fragments. For intermediate fixation IMF was maintained for 2 to 4 days for intermediate fixation and 4–6 weeks for definitive fragment fixation. For both intermediate and definitive MMF, self‐drilling intermaxillary fixation screws (OmniMax MMF System, Zimmer Biomed) were used. In patients presenting multiple (comminuted) fractures, the concomitant fractures (corpus/body) were also treated with internal fixation using miniplate osteosynthesis.

For all patients included, early (within 4 weeks) and late (after 6 months) complications were evaluated at the 1‐ and 6‐month postoperative follow‐ups. Early complications included assessment of: (a) malfusion, (b) wound infections, (c) wound dehiscence, (d) plate loosening, and (e) significant occlusal discrepancies. Late complications included (a) persistent occlusal disorders, (b) surgically induced (iatrogenic) transient facial nerve palsy, and (c) hypertrophic postoperative scarring.

### 2.5 Data Analysis and Statistics

Statistical analysis of epidemiologic and patient‐related factors was performed using Fisher's exact test for categorical variables due to the small sample size of mandibular ramus fractures. To evaluate the impact of injury mechanisms on fracture patterns, we employed Pearson's Chi‐squared tests and Fisher's exact tests. To pinpoint the source of significance, standardized residuals were analyzed, with values exceeding ±2.0 considered significant. Furthermore, the risk of sustaining multiple fractures following an RTA was quantified using the odds ratio (OR) with a 95% confidence interval. *p*‐values < 0.05 were considered statistically significant.

## 3 Results

### 3.1 Epidemiologic Data Classification

Between the period of January 2020 and January 2025, out of 547 patients with diagnosed mandibular fractures, 15 patients met the inclusion criteria for the SG and presented with mandibular ramus fractures, accounting for 2.7% of all mandibular fractures. For comparison, a CG consisting of 115 patients (21%) with mandibular angle fractures was enrolled.

Epidemiologic data, as well as patient‐ and surgery‐related factors for both the SG and CG are presented in Table 1. Although age did not differ (*p* = 0.742) between patients with mandibular ramus fractures (36.2 ± 6.2 years) and those with mandibular angle fractures (37.9 ± 19.3 years), a statistically significant difference was observed in gender distribution (*p* = 0.021, Fisher's Exact Test). While the vast majority of patients with mandibular angle fractures were male (90.4%), the proportion of female patients was significantly higher in the ramus fracture group (33.3% vs. 9.6%, *p* = 0.021).

**Table 1:** Epidemiologic and patient‐ and surgical‐related characteristics of mandibular ramus fractures (study group, SG) compared with mandibular angle fractures (control group, CG).

Variable	SG: Ramus fracture (*n* = 15)	CG: Angle fracture (*n* = 115)	*p*‐value
Age, mean ± SD (years)	36.2 ± 6.2	37.9 ± 19.3	0.742^a^
Sex, female/male, *n* (%)	5/10 (33.3/66.7)	104/11 (90.4/9.6)	0.021^b^
Fracture pattern, *n* (%)			
Singular	5 (33.3)	68 (59.1)	0.042^b^
Multiple (2 sites)	10 (66.6)	43 (37.4)	—
Multiple (3 sites)	0 (0)	4 (3.4)	—
Etiology, *n* (%)			
Violence	3 (20.0)	41 (35.6)	0.003^c^
Fall	2 (13.3)	44 (38.2)	—
Road traffic accident	7 (46.6)	13 (11.3)	—
Other accidents	3 (20.0)	17 (14.8)	—
Third molar present, *n* (%)	9 (60.0)	83 (72.2)	0.37^b^
Gonial angle, mean ± SD (°)	130.3 ± 7.2	127.4 ± 6.2	0.098^a^
Treatment, *n* (%)			
ORIF	13 (86.6)	115 (100)	0.012^b^
MMF	2 (13.4)	0 (0)	—

Abbreviations: CG, control group; MMF, maxillomandibular fixation; ORIF, open reduction and internal fixation; SG, study group.

^a^ Independent‐samples *t*‐test.

^b^ Fisher's exact test.

^c^ Chi‐square test.

Among the evaluated ramus fractures, 5 of 15 (33.3%) were isolated fractures whereas a clear majority of 10 of 15 (66%) presented with accompanying additional (complex‐) fractures (Table 1; Figure 1). A significant correlation was observed between the anatomical location of the injury and fracture complexity. While fractures of the mandibular angle were predominantly singular (59.1%), fractures involving the ramus were significantly (*p* = 0.042, Fisher's Exact Test) more likely to be part of a multiple fracture pattern (66%). The odds ratio (OR) of 2.91 indicates that ramus involvement nearly triples the likelihood of a multi‐fragment injury compared to angle fractures.

**Figure 1:**
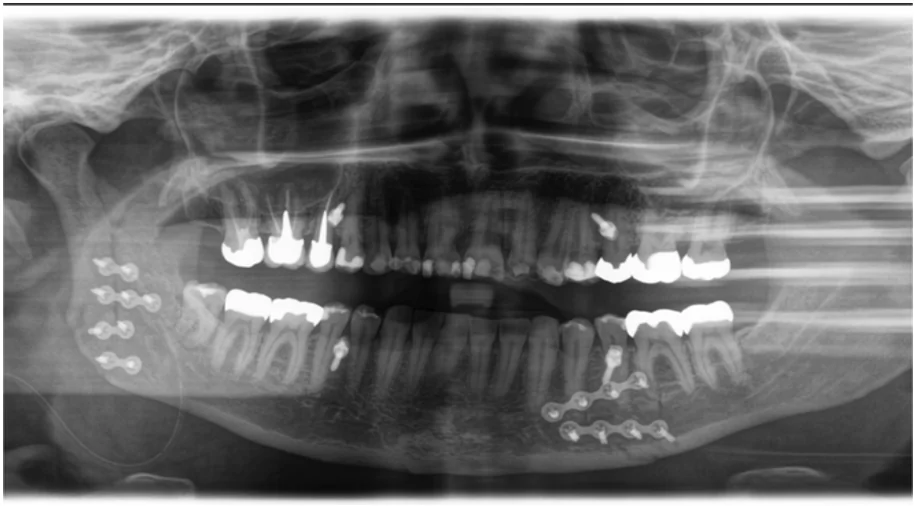
Present vertical mandibular ramus fracture extending from the sigmoid notch to the mandibular gonion combined with a contralateral body fracture.

According to the Agarwal and Mehrotra (2020) classification, only Type I (80%) and Type V (20%) fractures were observed. In detail, 12 of 15 ramus fractures presented as Type I (Figure 1; vertical/oblique fracture line extending from the sigmoid notch to either the inferior border or angle of mandible) and 3 of 15 patients presented Type V (comminuted fracture of ramus of mandible).

### 3.2 Trauma Mechanism and Injury Severity

Regarding the etiology of mandibular ramus fractures, road traffic accidents (RTAs) were the most common cause, occurring in 7 of 15 patients (46.6%), followed by violence in 3 of 15 (20%), domestic/sports accidents in 3 of 5 (20%), and falls in 2 of 15 (13.3%, Table 1). Compared to mandibular angle fractures, the distribution of trauma mechanisms differed significantly (*p* < 0.025), representing mandibular ramus fractures predominantly caused by RTAs, whereas angle fractures were more frequently associated with interpersonal violence (35.6%) and falls (38.2%; Tables 1, 2).

**Table 2:** Correlation between injury mechanism and fracture severity for mandibular ramus and angle fractures combined (*n* = 130).

Injury mechanism	Singular fracture (*n* = 73)	Multiple fracture (*n* = 57)	Total (*n* = 130)	Standardized residual (Multiple)	Odds ratio (95% CI)	*p*‐value
RTA	4 (20.0)	16 (80.0)	20	+3.24	6.48 (1.98–21.2)	< 0.001
Violence	26 (59.1)	18 (40.9)	44	−1.21	0.82 (0.39–1.74)	0.124
Fall	30 (65.2)	16 (34.8)	46	−2.01	0.53 (0.25–1.12)	0.043
Accidents	13 (65.0)	7 (35.0)	20	−1.26	0.65 (0.23–1.78)	0.138

*Note:* Values are presented as absolute numbers and row percentages (%). Statistical significance was assessed using Pearson's chi‐square test (χ^2^ = 16.58, df = 3, *p* = 0.0009). Standardized residuals > |1.96| indicate a significant deviation from the expected distribution.

Abbreviation: RTA, road traffic accident.

Table 2 illustrates the impact of the trauma mechanism on the number of fracture lines for all (15 ramus, 115 angle) mandibular fractures (*n* = 130) evaluated. The synthesis of standardized residuals reveals two distinct traumatic profiles within the study population. In this context, Road Traffic Accidents (RTA) showed the highest correlation with multiple (complex) fractures, as evidenced by a significant positive standardized residual of +3.24 (*p* < 0.001; Table 2). Interestingly, Road Traffic Accidents (RTA) acted as a potent predictor for complex injuries, increasing the risk of multiple fractures by a factor of 6.48 (OR 6.48, 95% CI 1.98–21.21, Table 2).

In contrast, a significant reciprocal trend was identified for falls; this mechanism was predominantly associated with singular fracture lines, showing a reduced likelihood for multiple fractures (OR = 0.53, *z* = −2.01; Table 2). Interpersonal violence and general accidents also showed no statistically significant directional preference, although their odds ratios (0.82 and 0.65, respectively) leaned toward simpler fracture patterns (Table 2).

### 3.3 Risk Factor Analysis

Table 3 details the presence and impaction depth characteristics of mandibular third molars in both the mandibular ramus (*n* = 15) and mandibular angle fracture (*n* = 115). While the overall presence of third molars did not differ significantly between the groups (SG: 9/15 [60%], CG:83/115 [72.1%]; *p* = 0.366), their eruption status correlated significantly with the fracture site. At the patient level, fractures of the mandibular angle were significantly more often associated with deeply impacted third molars (38.3%) compared to ramus fractures (13.3%; *p* = 0.049, Table 3).

**Table 3:** Number and characteristics of mandibular third molars in the mandibular ramus fracture (study group, SG; *n* = 15) and mandibular angle fracture (control group, CG; *n* = 115) groups.

PGC class	Mandibular ramus fractures (SG; *n* = 15)				Mandibular angle fractures (CG; *n* = 115)			
	A	B	C	Total	A	B	C	Total
1	4 (44.4)	0	0	4 (44.4)	20 (24.1)	6 (7.2)	1 (1.2)	27 (32.5)
2	3 (33.3)	0	1 (11.1)	4 (44.4)	13 (15.7)	12 (14.4)	4 (4.8)	29 (34.9)
3	0	0	1 (11.1)	1 (11.1)	4 (4.8)	7 (8.4)	16 (19.2)	27 (32.5)
Total	7 (77.7)	0	2 (22.2)	9 (100)	37 (44.6)	25 (30.1)	21 (25.3)	83 (100)

Comparison of third molar prevalence and impaction depth between groups			
Variable	SG, *n*/*N* (%)	CG, *n*/*N* (%)	*p*‐value
No third molar present	6/15 (40.0)	32/115 (27.8)	—
Third molar present	9/15 (60.0)	83/115 (72.2)	0.366
Deep impaction, per patient	2/15 (13.3)	44/115 (38.3)	0.049
Deep impaction, per molar	2/9 (22.2)	44/83 (53.1)	0.105

*Note:* Values are *n* (%) of third molars within each group.

Abbreviations: CG, control group; DI, deep impaction; PGC, Pell and Gregory classification (class 1–3, position A–C); SG, study group.

Within the subgroup of patients with present third molars (*n* = 92), a notable trend regarding impaction depth was observed. In the angle fracture group, 53.1% (44/83) of third molars were deeply impacted, compared to only 22.2% (2/9) in the ramus fracture group. While this difference did not reach full statistical significance (*p* = 0.105, Fisher's exact test), the odds ratio of 0.25 indicates that deep impaction is substantially less frequent in cases involving the mandibular ramus (Table 3).

The mean gonial angle in patients with ramus fractures was (130.3° ± 7.2°) compared to (127.4° ± 6.2°) in the angle fracture group without any statistical significance (Table 1; *p* = 0.098, Student's *t*‐test). However, a significant correlation was observed between mandibular morphology (gonial angle) and the eruption status of third molars. Patients with deeply impacted third molars (*n* = 46)—predominantly found in the mandibular angle group—exhibited a significantly more acute mean gonial angle (127.1° ± 6.1°) compared to those with erupted or absent teeth (*n* = 84;129.8° ± 6.9°, *p* = 0.028).

### 3.4 Treatment—Clinical Outcome

Thirteen of 15 (86.6%) patients were managed by open reduction and internal fixation (ORIF), which was significantly more frequently done than closed reduction (2/15; 13.4%, *p* < 0.01; Figures 1 and 2a; Table 4). Patients undergoing open reduction had supplemental maxillomandibular fixation for only 2–4 days (Figures 1 and 2a). Closed reduction using maxillomandibular fixation (MMF) for 6–8 weeks was performed in only two patients (Type V) who presented with anatomically favorable fracture lines with minimal displacement (Table 4). Detailed individual (*n* = 15) epidemiologic‐, patient‐ and surgical‐related factors as well as treatment modality used are listed in Table 4.

**Figure 2:**
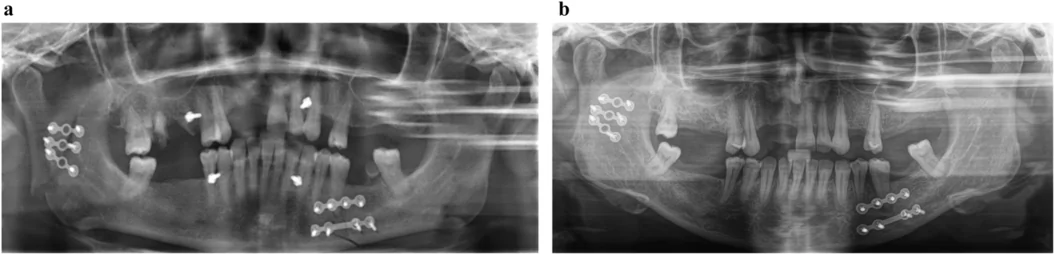
(a/b) Oblique vertical mandibular ramus fracture extending from sigmoid notch to the posterior border of the mandibular ramus. (a) immediate postoperative; (b) healed situation.

**Table 4:** Individual epidemiologic, patient‐, and surgical‐related factors for the mandibular ramus fractures evaluated (study group; *n* = 15).

No.	Sex	Cause of injury	Fracture	Type	PGC	Concomitant fracture	Treatment
1	M	Violence	Multiple	1	C3	Paramedian	ORIF
2	F	RTA	Multiple	5	A1	Paramedian	ORIF
3	F	RTA	Singular	5	—	—	ORIF
4	F	RTA	Multiple	1	—	Paramedian	ORIF
5	M	RTA	Singular	1	—	—	ORIF
6	M	Accident	Multiple	1	—	Corpus	ORIF
7	F	Fall	Multiple	1	A1	Corpus	ORIF
8	M	Accident	Multiple	1	A2	Corpus	ORIF
9	F	Violence	Multiple	1	A1	Paramedian	ORIF
10	M	Accident	Multiple	5	—	Corpus	MMF
11	M	RTA	Multiple	1	—	Corpus	ORIF
12	M	RTA	Multiple	1	A2	Paramedian	ORIF
13	M	Fall	Singular	1	C2	—	ORIF
14	M	Violence	Singular	1	A2	—	MMF
15	M	RTA	Singular	1	A1	—	ORIF

Abbreviations: F, female; M, male; MMF, maxillomandibular fixation; ORIF, open reduction and internal fixation; PGC, Pell and Gregory classification of the third molar (—, no third molar present in the fracture line); RTA, road traffic accident.

Early complications of ORIF included mild occlusal discrepancy (1×) and wound infection in one patient. Early visible transient marginal facial nerve palsy on the operated side occurred in one patient and recovered completely by the 6‐month evaluation. Transient facial nerve palsy was related to hook placement during clinical assistance. Conservative dental treatment assisted in the therapy for the malocclusion. All fractures healed successfully. Even if there were minor inaccuracies at the ramus margin (Figure 2b) representing some harmless exostosis or callus formation, no malocclusion was induced.

## 4 Discussion

Although a variable incidence of mandibular ramus fractures has been reported in numerous studies, the prevalence of ramus fracture of 2.7% presented in the current evaluation is in accordance with the range of previous accounts (Ellis et al. 1985; Agarwal et al. 2017; Olson et al. 1982; Bonitz et al. 2025). However, the disparities and different observed incidence of mandibular ramus fractures can be attributed to varying structural delineations of fracture lines in this region (Agarwal et al. 2017; Agarwal and Mehrotra 2020). In addition, overlapping features, such as concurrent fractures of the condyle, the subcondylar region, the coronoid, and even of the angle fracture, might preclude a clear ramus fracture diagnosis (Jadav et al. 2015; Duan and Zhang 2008).

In contrast to the previous findings of Jadhav et al. (2015) reporting an incidence of only 8.3%, the present study showed a substantially higher rate of isolated ramus fractures at 33.3%. Consistent with the reports of Jadhav et al. (2015) and Agarwal and Mehrotra (2020), the majority of the ramus fractures in the present cohort occurred in conjunction with other associated mandibular fractures. However, contrary to the findings of Jadhav et al. (2015), showing no correlation with the typical pattern of associated fractures, the present findings demonstrate evident similarities among adjunctive fractures. In this context, 90% of mandibular ramus fractures associated with other mandibular fractures involved fractures of the contralateral/ipsilateral or corpus/paramedian region.

Considering the etiologic factors, predominantly road traffic accidents (46.6%) with direct force application were found to be the main reasons generating ramus fractures. These findings are in accordance with the studies of Jadhav et al. (2015) and Agarwal and Mehrotra (2020) presenting road traffic accidents with an incidence of 58.3% and 75.7%, respectively, as the primary cause, followed by falls with 33.3% or 15.2%, respectively. According to the findings of the present study reporting a significant correlation between the injury mechanism and fracture severity (*p* < 0.001), the current data identifies road traffic accidents (RTA) as the primary predictor for complex/multiple fracture patterns. With a significant standardized residual of +3.24 and a high odds ratio (OR = 6.48), patients involved in an RTA were more than six times as likely to sustain multiple fractures compared to other mechanisms. Biomechanically, this observation might be explained by the high kinetic energy transferred during a vehicular impact (Unnewehr et al. 2003). Unlike low‐velocity trauma, such as falls or interpersonal violence, which typically act as focal forces resulting in singular “locus minoris resistentiae” failures (reciprocal trend for falls: OR 0.53), the blunt force in RTA is often distributed across the entire mandibular arch (Unnewehr et al. 2003; Boffano et al. 2015; Giannunzio et al. 2026). This leads to a systemic failure of the bone at multiple points, frequently involving the ramus, which has been identified as a site requiring higher energy to fracture (Unnewehr et al. 2003; Giannunzio et al. 2026). In addition, the difference of gender distribution between the prevalence of mandibular ramus and mandibular angle fracture might be attributed to different prevalence of fracture causality with lesser interpersonal violence for ramus fractures. Consequently, the significantly higher rate of multiple fractures in mandibular ramus fractures can be attributed directly to the higher proportion of RTA cases irrespective of sex (3 f/4 m; 46.6%). Subsequently, these findings suggest that clinicians should maintain a higher index of suspicion for secondary fracture lines when a high‐energy RTA mechanism is reported, particularly in the ramus region (Boffano et al. 2015; Giannunzio et al. 2026).

Regarding the fracture morphology, a specific fracture pattern was observed within the ramus group according to the Agarwal and Mehrotra (2020) classification. Despite the high kinetic energy involved in RTAs, 80% of ramus fractures (12/15) were classified as Type I (simple linear fractures), while only 20% (3/15) presented as comminuted Type V fractures. This suggests that the robust soft tissue envelope, specifically the muscular splinting provided by the masseter–pterygoid sling, stabilizes the ramus during impact (Giannunzio et al. 2026; Kroon et al. 1991). Even when the total energy is sufficient to cause multiple mandibular fractures, this stabilization tends to limit displacement and fragmentation within the ramus itself, leading to the observed predominance of Type I injuries (Kroon et al. 1991).

Interestingly, the present study also evaluated the influence of mandibular third molars on the generation of mandibular ramus fractures (Alfertshofer et al. 2025; Giovacchini et al. 2018; Kandel et al. 2021). As reported in numerous previous studies, the prevalence and location of third molars have been attributed to weakening the mandibular angle and influencing the formation of mandibular angular fractures (Kandel et al. 2021; Armond et al. 2017; Zhu et al. 2005). Meta‐analyses conducted by Giovacchini et al. (2018) and Kandel et al.(2021) report in detail about the association between the presence of lower third molars and mandibular angle fractures, demonstrating that the likelihood of angular fractures increases with greater impaction depth in both the vertical (position A–C) and horizontal (classes I–III) directions. Nevertheless, there is a discrepancy in the literature as to whether mandibular resistance depends on cortical bone layer characteristics, such as thickness, density, and continuity. In their biomechanical analyses, Rudderman et al. (2008) and Bezerra et al.(2013) provided evidence that muscle insertions and occlusion create an area of tension at the superior border of the mandibular angle (i.e., the oblique ridge) and compression at the lower margin of the angular region (Rahimi‐Nedjat et al. 2016). Numerous investigations have reported consistent results showing that the presence and position of the lower third molars may influence the site of mandibular fractures and subsequently, if the continuity of the cortical ridge is disrupted by a third molar, the mandible may be more susceptible to angle fractures (Rahimi‐Nedjat et al. 2016; Alfertshofer et al. 2025). Conversely, it has been reported that in the absence of a third molar, the mandibular angle region exhibits greater structural continuity, allowing forces originating from the symphysis to be transmitted through the angle to the ramus and condyle, potentially resulting in condylar and ramus fractures (Sawazaki et al. 2010; Antic et al.^2016^). Therefore, when an impacted lower third molar is absent (i.e., totally erupted or missing), the mechanical stress may be concentrated on the posterior and buccal part of the condylar process, which may increase the risk of condylar fractures (Sawazaki et al. 2010; Rahimi‐Nedjat et al. 2016; Alfertshofer et al. 2025; Zhao et al. 2023).

In the present evaluation, the presence of the third molar was initially categorized according to the Pell and Gregory classification and was subsequently subdivided into deep impaction versus erupted/absent. By using this binomial rating additionally to evaluate its influence on the occurrence of mandibular ramus fractures, the findings demonstrated that 86.7% of the ramus fractures occurred in the absence of deeply impacted third molars. Subsequently, these findings allow us to conclude that, contrary to angle fractures where deep impaction acts as a localized stress riser, mandibular third molars were not significantly involved in the pathogenesis of mandibular ramus fractures (Alfertshofer et al. 2025). Instead, ramus fractures appear to occur independently of dental impaction depth, likely requiring higher kinetic energy to overcome the structural continuity of the posterior mandible (Meisami et al. 2002; Tevepaugh and Dodson 1995).

However, detailed data from previous studies have also reported that mandibular angle fractures are associated with a higher gonial angle than mandibular body, symphysis, and condylar fractures (Al‐Dawoody 2022; Dhara et al. 2019; Xiao et al. 2011; Abdul Rehman et al. 2020). It has been reported that the gonial angle determines bone thickness via muscle attachment, with a larger gonial angle representing a thinner cortical bone architecture that might be associated with a higher prevalence of mandibular angle fractures (Panneerselvam et al. 2022; Bereznyak Elias et al. 2018). Therefore, a deep third molar impaction in conjunction with the presence of a high gonial angle may be two primary factors promoting the formation of mandibular angle fractures (Kandel et al. 2021; Antic et al. 2016; Al‐Dawoody 2022).

According to the present findings, mandibular ramus fractures were also in association with a high gonial angle (Junior et al. 2019; Arora et al. 2025). In this context, a large gonial angle without the presence of deeply impacted third molars might be a predominant combination predisposing to mandibular ramus fractures (Alfertshofer et al. 2025; Rahimi‐Nedjat et al. 2016). The observed correlation between a larger gonial angle (130°) and a decreased impaction depth in the mandibular ramus fracture group can be explained by the increased anatomical space within the retromolar area (Rahimi‐Nedjat et al. 2016). A more obtuse gonial angle typically results in a more distal inclination of the ascending ramus, providing sufficient space to facilitate the vertical eruption of third molars. This effectively reduces the likelihood of deep bony impaction and, consequently, minimizes the structural weakening of the mandibular angle, thereby shifting mechanical stress to the ramus when high‐energy trauma occurs (Bezerra et al. 2013; Rahimi‐Nedjat et al. 2016).

Given the similarities in the radiographic measurements of the gonial angle between mandibular angle and ramus fractures, their morphological relationship warrants further discussion. This is supported by the findings of Arora et al. (2025), who reported a mean gonial angle of 127.8° ± 6.9° for angle fractures—a value significantly higher than those found in fractures of the condyle (124.5°), body (121.3°), and symphysis (124.0°). In light of the present findings and the data from Arora et al. (2025), it is reasonable to question whether ramus fracture truly represents an independent clinical entity or should instead be defined as a sub‐classification of mandibular angle fractures. Both locations share a similar morphological baseline (obtuse gonial angle), with the specific fracture site potentially determined by secondary factors such as the presence or impaction depth of third molars (Arora et al. 2025; Dhara et al. 2019).

The management of mandibular ramus fractures remains controversial, with treatment decisions often driven by fracture displacement and the degree of an open bite rather than strict guidelines. While both open reduction and internal fixation (ORIF) and closed treatment start with intermaxillary fixation, ORIF offers faster functional rehabilitation and significantly shorter fixation times compared to conventional closed reduction, resulting in high success rates (Alfertshofer et al. 2025; Kim et al. 2022; Nandini et al. 2011).

According to the present findings, both ORIF and closed maxillomandibular fixation (MMF) for mandibular ramus fractures yield excellent clinical outcomes, with ORIF offering the advantage of early functional rehabilitation and significantly reduced fixation time (3 days vs. 6 weeks). The findings of the present study, in which 86.7% of patients were successfully treated via intraoral ORIF, conclude that both methods are effective, but ORIF facilitates faster recovery, improved nutrition, and better oral hygiene (Kim et al. 2022; Nandini et al. 2011). As both ORIF and closed MMF yielded excellent clinical outcomes, a satisfactory result can be anticipated for mandibular ramus fractures regardless of the chosen method (Kim et al. 2022; Nandini et al. 2011).

Concerning the limitations of this study, the retrospective, single‐center study design restricts the generalizability of the findings. Additionally, anatomical overlaps complicate the distinction from condylar or angle fractures, and the morphological sub‐analyses are limited by small subgroup sizes. Furthermore, the stabilizing effect of the masseter–pterygoid sling was only indirectly inferred rather than functionally or biomechanically quantified. Lastly, the clinical comparison between ORIF and MMF lacks a randomized controlled design, as treatment selection was directly driven by fracture displacement and the presence of an open bite.

## 5 Conclusion

Mandibular ramus fractures representing a prevalence of 2.7% are primarily caused by high‐velocity road traffic accidents, serving as a strong predictor for complex multiple fracture patterns (OR = 6.48). A simple linear vertical fracture pattern dominates in 80% of cases, which may be attributed to the stabilizing effect of the masseter–pterygoid muscle sling. The anatomical combination of a large gonial angle and the absence of deeply impacted third molars predisposes patients to fractures in the ramus region. While both ORIF and closed MMF yield excellent outcomes, intraoral ORIF offers superior functional rehabilitation with significantly shorter fixation times and earlier recovery.

## Author Contributions

**Stefan Krennmair:** concept/design, analysis, statistics, writing. **Simon Kienberger:** data collection, data analysis. **Matthias Angerer:** data analysis, statistics. **Lukas Postl:** concept, discussion. **Gerald Krennmair:** drafting, discussion, interpretation. **Michael Malek:** concept/design, data interpretation, analysis, revision.

## Funding

The authors have nothing to report.

## Ethics Statement

The study protocol was approved by the local ethics committee (JKU: #. 1485/2025).

## Conflicts of Interest

The authors declare no conflicts of interest.

## Data Availability

The data that support the findings of this study are available from the corresponding author upon reasonable request.
